# PPARs and Xenobiotic-Induced Adverse Effects: Relevance to Human Health

**DOI:** 10.1155/2010/954639

**Published:** 2011-06-07

**Authors:** Christopher Lau, Barbara D. Abbott, J. Christopher Corton, Michael L. Cunningham

**Affiliations:** ^1^Toxicity Assessment Division, National Health and Environmental Effects Research Laboratory, Office of Research and Development, US Environmental Protection Agency, Research Triangle Park, NC 27711, USA; ^2^Integrated Systems Toxicology Division, National Health and Environmental Effects Research Laboratory, Office of Research and Development, US Environmental Protection Agency, Research Triangle Park, NC 27711, USA; ^3^National Toxicology Program, National Institute of Environmental Health Sciences, National Institute of Health, Research Triangle Park, NC 27709, USA

The peroxisome proliferator-activated receptors (PPARs) are members of the nuclear receptor superfamily that act as transcription factors and play important roles in the regulation of a variety of biological processes, such as adipocyte proliferation and differentiation, glucose homeostasis, intracellular trafficking of lipids and their metabolism, inflammatory responses, vascular functions, and embryonic and fetal development. Three PPAR subtypes have been identified: PPAR*α*, PPAR*β*/*δ*, and PPAR*γ* (with isoforms PPAR*γ*1 and PPAR*γ*2), each with overlapping but unique ligand specificity, patterns of tissue distribution, and biological functions. The mechanisms of PPAR action have been well studied [1]. The nuclear receptors are activated by their ligands, heterodimerize with another nuclear receptor, retinoid X receptor (RXR), and undergo specific conformational changes that release corepressors and allow for recruitment of coactivators. The receptor complex binds to specific DNA sequences, called peroxisome proliferator response elements (PPREs), in the promoter regions of target genes for transactivation as well as transrepression. Activated genes are associated with fatty acid transport and metabolism, adipogenesis, peroxisome biogenesis, cholesterol and bile acid biosynthesis, proteasome activation, and glucose metabolism; repressed genes typically include those involved with adaptive inflammatory responses.

A number of endogenous ligands have been identified for each PPAR subtype and include long-chain polyunsaturated fatty acids such as linoleic and arachidonic acids, saturated fatty acids, and eicosanoids. Because of the involvement of PPARs in controlling energy homeostasis, synthetic chemicals have been designed to interact with these nuclear receptors for therapeutic intervention of a number of metabolic diseases such as obesity, type-2 diabetes, and atherosclerosis. Indeed, in the past several decades, specific pharmacologic agents such as the fibrates (that include clofibrate, fenofibrate, ciprofibrate, bezafibrate, and gemfibrozil) and the glitazones (such as ciglitazone, troglitazone, rosiglitazone and pioglitazone) have been developed that target PPAR*α* and PPAR*γ*, respectively, for the effective treatment of hyperlipidemia and diabetes. Since 1990 when the PPAR family members were cloned and characterized, a number of industrial and consumer chemicals, pesticides, and environmental contaminants have been shown to activate PPARs. These include di-(2-ethylhexyl)phthalate (DEPH) [2], diisobutyl phthalate [3], trichloroethylene, di- and trichloroacetic acids, [4], bisphenol A [5], butylparaben [3], perfluoroalkyl acids (PFAAs) [6], and organotins [7]. Systematic screening of chemicals in commerce and in the environment for PPAR molecular signature and functional activities may further expand the existing list [8–10]. However, the potential human and ecological health risks from such chemically induced PPAR activation are still relatively unknown and presently subject to great debate. 

This special issue is organized to highlight the recent advances made in identifying drugs and chemicals that target PPARs as their mechanism-of-action, in characterizing the downstream biochemical and physiological consequences from these drug actions and chemical insults, and in addressing the relevance of this mechanism-of-action and toxicity for human health risks. This issue will focus on both cancer and noncancer effects (that include reproductive, developmental, immunologic and metabolic endpoints), and unique actions mediated by the different PPAR subtypes. 

There are eight papers in this special issue, including five original research articles and three reviews. In the first research article, “*Peroxisome proliferator-activated receptors alpha, beta, and gamma mRNA and protein expression human fetal tissues*,” B. D. Abbott et al. characterize the mRNA and protein expression of the three PPAR subtypes in human fetal tissues. With the exception of one study that previously described the expression of PPAR proteins in the human fetal digestive tract, this is the first comprehensive report to compare the expression of these nuclear receptor subtypes in human fetal liver, heart, lung, kidney, stomach, intestine, adrenal, spleen and thymus during organogenesis, and to contrast the levels of expression in the fetus to those in adult tissues. This study reports that PPAR*α*, *β* and *γ* were expressed in all nine human fetal tissues evaluated. In general, mRNA expression of PPAR subtypes varied by tissue; notably, the levels in fetus were comparable to or even higher than those in adult, a pattern similar to that observed in rodents. These findings indicate that PPARs likely serve key roles in regulating developmental events, and inappropriate or untimely activation of these nuclear receptors (through transplacental delivery of drugs or exposure to xenobiotic chemicals) may bear untoward health consequences. Subsequent to the appearance of this paper online, these investigators discovered an artifact in their detection for PPAR*γ* proteins, and have conducted an additional study to rectify the unexpected error. The replacement findings have now been published in “*Erratum to “Peroxisome proliferator activated receptors alpha, beta, and gamma mRNA and protein expression in human fetal tissues,”*” and the new protein results are in good agreement with the patterns of expression obtained for PPAR*γ* mRNA in these tissues. 

In a review article entitled “*The role of PPAR*α* activation in liver and muscle*,” L. Burri et al. summarize the involvement of PPAR*α* in two metabolically active tissues, liver and skeletal muscle, and provide a comparative overview of the benefits and risks of PPAR*α* activation in humans and rodents. The beneficial effects of PPAR*α* activation in counteracting metabolic disorders are well supported in both animal and human studies. Indeed, both species share multiple changes in expression of genes that belong to functional classes related to lipid metabolism. Yet, there are substantial differences between human and mouse target gene expression in response to PPAR*α* activation in the liver, particularly those associated with peroxisome proliferation, hypertrophy, hyperplasia, apoptosis and tumor induction. The responses to PPAR*α* activation appear to be more pronounced in mice than in humans. In contrast to mice, humans show no effect on glucose metabolism in response to PPAR*α* activation; conversely, apolipoprotein production that leads to a decrease of VLDL and an increase of HDL cholesterol is only seen in humans treated with a PPAR*α* activator. PPARs are expressed in skeletal muscles in humans and rats; activation of these nuclear receptors increases lipid oxidation and decreases triglyceride accumulation and alters glucose metabolism. These investigators note a sex difference in both humans and rodents in response to PPAR*α* activation and caution that gender differences should be taken into consideration for therapy involving PPARs.

The theme of sex differences related to PPAR*α* effects is continued in a second review article presented by M. Yoon “*PPAR*α* in obesity: sex difference and estrogen involvement*” who describes sexual dimorphism in the treatment of obesity by PPAR*α* ligands and summarizes the involvement of estrogen. Both PPAR*α* and estrogen receptors (ERs) are involved in regulating adiposity. Interestingly, PPAR/RXR heterodimers have been shown to bind to estrogen response elements, and PPARs and ERs share certain cofactors, suggesting that signal cross-talk between these two nuclear receptors may participate in the control of obesity. However, sex-related differences have been reported in PPAR*α* effects in animal studies. Fenofibrate reduced weight gain and adiposity in male mice given a high-fat diet and reduced circulating cholesterol and triglycerides, while females exhibit drug resistance. In fact, estrogens appear to inhibit PPAR*α* action on obesity. While both fenofibrate and estradiol (E2) by themselves were effective in attenuating weight gain and increases of fat mass in mice fed a high-fat diet, combined fenofibrate and E2 treatment did not produce any additional effects; the combined treatment actually led to elevated levels of circulating cholesterol and triglycerides compared to those with each treatment alone. Findings from animal studies are by and large in agreement with clinical observations. Details about the interplay between PPAR*α* and ERs are presently unavailable, but competition between these two nuclear receptors for transcriptional coactivators and corepressors may confer a negative cross-talk between their actions.

A research article by M. Cunningham et al. “*Effects of the PPAR*α* agonist and widely used antihyperlipidemic drug gemfibrozil on hepatic toxicity and lipid metabolism*” follows the discussion of the use of antihyperlipidemic drugs, focusing on lipid metabolism and hepatic toxicity of another fibrate, gemfibrozil, and comparing the responses between rats, mice, and hamsters. Gemfibrozil is a valuable therapeutic agent in the control of coronary heart disease, in part due to its hypolipidemic effects in reducing levels of triglycerides and LDL cholesterol and raising those of HDL. Similar to other peroxisome proliferators, gemfibrozil is known to induce liver hypertrophy and tumors in rodents. This paper summarizes the results of several studies conducted by the National Toxicology Program to evaluate the effects of chronic exposure to gemfibrozil in rats and mice; evaluation of hamsters is included because this species, like humans, is relatively resistant to the hepatotoxicity and carcinogenicity of peroxisome proliferators. In general, hepatic effects of gemfibrozil were seen in all three species, although rats appeared to be most responsive and hamsters to be least responsive. Correspondingly, a similar rank order of species difference was noted in the oxidative stress-related mechanisms-of-action produced by gemfibrozil, which may be related to the differential susceptibility to the hepatocarcinogenicity of this drug. Information provided in this paper should lend support in differentiating the beneficial effects of PPAR*α* drugs in treating dyslipidemia and their potential risks of tumor induction. 

Two research articles by C. J. Wolf et al., “*Developmental effects of perfluorononanoic acid in the mouse are dependent on peroxisome proliferator-activated receptor-alpha*,” and M. B. Rosen et al., “*Gene expression profiling in wild-type and PPAR*α*-null mice exposed to perfluorooctane sulfonate reveals PPAR*α*-independent effects*,” address the potential human health risks of perfluoroalkyl acids, a class of persistent environmental contaminants that has received intense scrutiny from regulatory agencies worldwide. PFAAs are found ubiquitously in all environmental media, distributed globally, present in humans and wildlife, and associated with several adverse effects in laboratory animal models. These chemicals vary in carbon-chain lengths and functional groups (chiefly carboxylates and sulfonates), but all appear to activate mouse and human PPAR*α* [6]. PPAR*α* activation by PFAAs has been shown previously to be related to their hepatotoxicity, developmental toxicity, and immunotoxicity in rodents. Results from previous studies with transgenic PPAR*α*-null mice have indicated that developmental toxicity of perfluorooctanoate (PFOA), but not that of perfluororooctane sulfonate (PFOS), is dependent on PPAR*α* [11, 12]. Using a similar experimental design, C. J. Wolf et al. in “*Developmental effects of perfluorononanoic acid in the mouse are dependent on peroxisome proliferator-activated receptor-alpha*” report that the adverse developmental effects of perfluorononanoic acid (PFNA) were more pronounced than those of PFOA, but also dependent on a PPAR*α* mechanism. Thus, neonatal mortality (at high doses), growth impairment and developmental delays (at lower doses) were observed in wild-type mice but not in PPAR*α*-null mice after gestational exposure to PFNA. These results therefore confirm a different mode-of-action for developmental effects between the perfluoroalkyl carboxylates and perfluoroalkyl sulfonates, and that the chemical potency of PFAAs increases with carbon-chain length. In contrast, the phenotypic responses in the liver of mice exposed to PFOA or PFOS are quite similar. Both fluorochemicals activate PPAR*α* and its target genes, inducing peroxisome proliferation, hypertrophy and tumors in the liver. However, M. B. Rosen and coworkers in “*Gene expression profiling in wild-type and PPAR*α*-null mice exposed to perfluorooctane sulfonate reveals PPAR*α*-independent effects*” report a number of genomic changes associated with lipid metabolism, inflammation and xenobiotic metabolism that are independent of PPAR*α* activation; rather, these gene expressions may be related to PPAR*γ*, PPAR*β*, or another nuclear receptor, the constitutive androstane receptor (CAR), thus indicating the possibility of multiple modes-of-action for PFAA hepatic effects. In addition, altered expression of certain genes unique to PFOS exposure was identified, including those associated with ribosome biogenesis, oxidative phosphorylation and cholesterol biosynthesis. These findings should provide valuable support for the assessment of human health risks of exposure to these environmental contaminants. 

Continuing the exploration of target genes activated by PPAR*α* and their attendant functional responses, H. Ren et al. in “*Regulation of proteome maintenance gene expression by activators of peroxisome proliferator-activated receptor*α**” focus on the regulation of proteome maintenance (PM) by this nuclear receptor. Increased oxidative stress caused by chemical or physical insult can lead to misfolding or other damage to protein, and restoration of cellular homeostasis entails stabilization of unfolded proteins by molecular chaperones (such as heat shock proteins, Hsp) or removal of damaged proteins by proteolysis. Ample evidence has suggested that PPAR*α* protects multiple tissues from oxidative stress induced by chemicals through altered expression of genes involved in proteome maintenance, including those in the Hsp family and proteasomal genes involved in proteolysis. These investigators compare and contrast the expression of PM genes with traditional target genes (e.g., lipid metabolizing enzymes) in rodent liver after exposure to seven diverse peroxisome proliferators (WY 14,643, fibrates, valproic acid, DEHP, and PFAAs). Genes and proteins involved in proteome maintenance were altered by these peroxisome proliferators, although the expression of many of these genes appeared to be delayed or transient, and was distinctly different from other typical PPAR*α*-dependent genes. These results therefore support an expanded role for PPAR*α* in regulating genes and proteins that serve as guardians of the proteome, in addition to controlling lipid metabolism and energy balance.

A. Rogue et al. in “*Gene expression changes induced by PPAR gamma agonists in animal and human liver*,” summarize the changes of hepatic gene expression induced by PPAR*γ* agonists in animal models and humans. PPAR*γ* is highly expressed in adipose tissues, and to a much lesser extent in the liver. PPAR*γ* drugs such as the glitazones are used to treat type-2 diabetes. They enhance insulin sensitivity presumably by channeling circulating fatty acids into adipose tissue. However, side effects of at least one of these agents include idiosyncratic hepatotoxicity, although the determinant factors for the untoward actions of PPAR*γ* agonists remain to be elucidated. The authors compare the gene expression profiles of PPAR*γ* activation derived from *in vivo* studies with rodent livers to those obtained from *in vitro* studies with rat and human hepatocytes. PPAR*γ* levels are enhanced in obese and diabetic mouse liver, and the steatogenic responses to glitazone in these rodent models are more pronounced than those seen in the lean controls. The genomic responses to PPAR*γ* agonists in the liver mirror the tissue distribution profile of this nuclear receptor; hence, only a small number of genes were affected in the liver compared to the adipose tissues. Only limited studies are available with human liver cells, and results from individual donors are quite variable, perhaps in line with the idiosyncratic nature of the hepatotoxicity observed. Future studies identifying specific PPAR*γ* genes in the liver will elucidate the etiology of hepatotoxicity associated with PPAR*γ* agonists, particularly after long-term therapeutic treatment.

In summary, this special issue provides a glimpse of the current understanding of PPAR involvement in therapeutic interventions, as well as the untoward side effects, and the potential health risks from exposure to xenobiotic chemicals found in the environment. These reviews and research papers contribute significantly to our understanding of these intriguing nuclear receptor signaling molecules. 
